# Isorhamnetin and Female Reproduction: Effects on Viability, Hormone Secretion, and Growth Factors

**DOI:** 10.3390/ph19081196

**Published:** 2026-07-30

**Authors:** Michal Mihal, Denis Bazany, Petr Slama, Adriana Kolesarova

**Affiliations:** 1 AgroBioTech Research Centre, Slovak University of Agriculture in Nitra, Tr. A. Hlinku 2, 949 76 Nitra, Slovakia; denis.bazany@uniag.sk; 2 Department of Animal Morphology, Physiology and Genetics, Faculty of AgriSciences, Mendel University in Brno, Zemědělská 1/1665, 613 00 Brno, Czech Republic; petr.slama@mendelu.cz; 3 Institute of Applied Biology, Faculty of Biotechnology and Food Sciences, Slovak University of Agriculture in Nitra, 949 76 Nitra, Slovakia

**Keywords:** isorhamnetin, reproduction, female, proliferation, apoptosis, cancer

## Abstract

**Background/Objectives**: Isorhamnetin is a naturally occurring flavonoid with reported antioxidant, anti-inflammatory, and anticancer properties. Although its biological activities have been extensively investigated, its effects on ovarian cell physiology remain insufficiently characterized. This study aimed to evaluate the influence of isorhamnetin on the viability, steroid hormone secretion, apoptosis, and growth factor signalling in human ovarian cell lines representing both non-tumour and tumour phenotypes. **Methods**: Human granulosa (HGL5), granulosa tumour (COV434), and epithelial ovarian carcinoma (OVCAR-3) cell lines were treated with isorhamnetin at concentrations of 5–80 μg/mL for 24 h. Cell viability was determined using the AlamarBlue^TM^ assay. The secretion of progesterone, 17β-estradiol, and the presence of transforming growth factor β2 (TGF-β2), transforming growth factor β receptor 2 (TGFBR2), and apoptosis-inducing factor (AIF) was quantified by enzyme-linked immunosorbent assay (ELISA). **Results**: Isorhamnetin significantly reduced cell viability in a dose-dependent manner, with tumour cell lines exhibiting greater sensitivity than non-tumour granulosa cells. A significant decrease in viability was observed in OVCAR-3 cells from 10 μg/mL onward, whereas COV434 cells showed significant reductions at concentrations of 20 μg/mL and higher. In contrast, HGL5 cell viability was significantly affected only at the highest concentration (80 μg/mL). No significant changes were detected in the secretion of progesterone, 17β-estradiol, TGF-β2, or TGFBR2 in any of the examined cell lines. A significant reduction in AIF production was observed only in HGL5 cells treated with 80 μg/mL isorhamnetin. **Conclusions**: Isorhamnetin preferentially decreased the viability of ovarian tumour cells while exerting only limited effects on non-tumour granulosa cells, indicating preferential cytotoxic activity toward malignant ovarian cells. The absence of significant changes in steroid hormone production and TGF-β suggests that its antiproliferative effects involve molecular pathways that were not investigated in the present study. These findings support further investigation of isorhamnetin as a potential natural compound for ovarian cancer prevention or adjunctive therapy.

## 1. Introduction

Reproductive health is greatly influenced by the interplay between hormones, oxidative stress, and environmental factors. Steroid hormones are important for regulating the function of the ovary, folliculogenesis, and maintenance of pregnancy and are also involved in the body’s antioxidant defence system [1,2]. Disruptions in the delicate balance may result in disease and disease states, such as ovarian dysfunction and malignancy. The increasing incidence of reproductive diseases and constitution-related ailments mandates the development of novel prevention and cure strategies. Phytonutrients, and, in particular, the flavonoids, have emerged as promising agents in modulating oxidative stress, cell proliferation, and apoptosis [3]. Among them are flavonoids such as quercetin and isorhamnetin [4,5]. Isorhamnetin, as a 3′-O-methylated derivative of quercetin, forms one of the main constituent flavonoids in sea buckthorn with proven pharmacological potential. Previous research has elucidated that isorhamnetin targets the PI3K/AKT/mTOR, MAPK, and NF-κB signalling systems, triggers apoptosis, and suppresses proliferation in carcinoma cells [6,7]. Further, its anticancer, anti-inflammatory, and cardioprotective activity underlines its importance for female reproductive wellness, where oxidative stress and aberrant apoptosis are pivotal [8,9]. Evidence indicates that isorhamnetin can inhibit cellular proliferation [9], induce apoptosis, reduce tumour progression [6,9,10], regulate inflammatory responses [7,9,11], enhance cognitive functioning [7], and influence several metabolic processes [11,12]. Despite these reports, studies on the immediate action of isorhamnetin in alleviating ovarian physiology are few. Some studies indicate that some of the flavonoids, such as quercetin, may influence granulosa cell proliferation, apoptosis, and secretion of hormones and may modulate gonadotropin activity [3,13]. However, the exact function of isorhamnetin in regulating viability, steroidogenesis, and apoptotic machineries within cells in the ovary has yet to be clearly elucidated. The present research aimed to investigate the effect of isorhamnetin on selected key parameters, such as viability, secretion of steroid hormones and growth factors, of female reproductive function in vitro, using the human granulosa cell line HGL5 and human ovarian carcinoma cell lines COV434 and OVCAR-3 as experimental models. The research focused on evaluating its potential role as a modulator of cellular viability, proliferation, and endocrine activity, thereby contributing to the understanding of its possible application in the prevention and therapy of ovarian pathologies. The present study was designed as an initial in vitro screening study to evaluate the biological activity of isorhamnetin in ovarian cell lines and to identify potential targets for future mechanistic investigations.

## 2. Results

The experimental part of the study was focused on the phytonutrient isorhamnetin. We assessed how isorhamnetin affected both tumour (COV434, OVCAR-3) and non-tumour (HGL5) cell lines, focusing particularly on viability processes, cell secretory activity (the release of growth factors and steroid hormones and the presence of their receptors), and the expression of markers of apoptosis.

### 2.1. Effect of Isorhamnetin on Cell Viability

**Figure 1:**
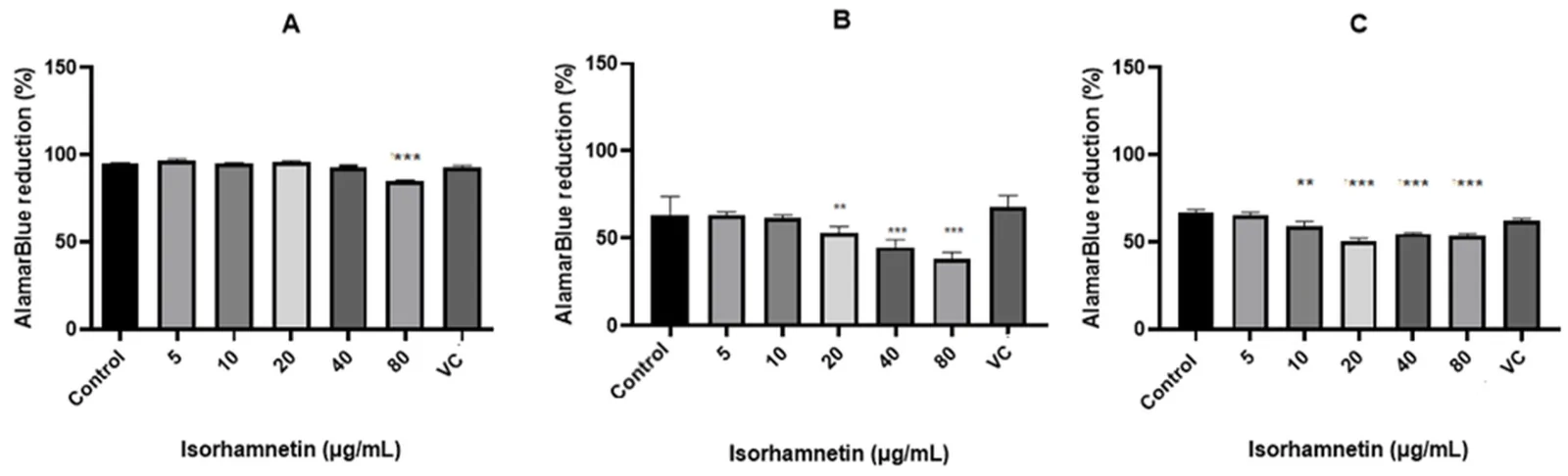
Viability of HGL5 (**A**), COV434 (**B**), and OVCAR-3 (**C**) cells after incubation with isorhamnetin (%). Cells cultured in medium without additives represent the control group (Control). Cells cultured in DMSO at a concentration corresponding to the highest applied concentration of the additive represent the vehicle control (VC). Other experimental groups represent cells with selected concentrations of isorhamnetin (5, 10, 20, 40, and 80 µg/mL) for 24 h. The data are expressed as mean values ± SEM. Significant differences between the control and experimental groups are at the level of ** (*p* ≤ 0.01) and *** (*p* ≤ 0.001). ELISA. AlamarBlue^TM^.

To investigate the effects of isorhamnetin on ovarian cell viability in vitro, non-tumour granulosa cells (HGL5), tumour granulosa cells (COV434) and epithelial ovarian carcinoma cells (OVCAR-3) were treated with different concentrations of isorhamnetin for 24 h. A significant decrease (*p* ≤ 0.001) in HGL5 cell viability was observed only at the highest concentration (80 µg/mL), while lower concentrations showed no significant effect. In contrast, a marked dose-dependent decrease in cell viability was detected in COV434 cells at concentrations of 20, 40, and 80 µg/mL (*p* ≤ 0.01–0.001) and in OVCAR-3 cells already from 10 µg/mL (*p* ≤ 0.01), with further reduction at higher concentrations (*p* ≤ 0.001). Compared to the control, tumour cell lines were significantly more sensitive to isorhamnetin than non-tumour cells (Figure 1).

To further characterize the cytotoxic potency of isorhamnetin, IC_50_ values were estimated from the viability data. In HGL5 non-tumour granulosa cells, cell viability remained above 50% throughout the tested concentration range; therefore, the IC_50_ value was estimated to be >80 µg/mL, indicating relatively low sensitivity to isorhamnetin. In contrast, COV434 granulosa tumour cells exhibited considerably higher sensitivity, with an estimated IC_50_ of approximately 26.3 µg/mL, consistent with the observed concentration-dependent reduction in viability. In OVCAR-3 cells, although isorhamnetin significantly reduced cell viability compared with the control, a monotonic concentration–response relationship was not observed at the highest tested concentrations. Therefore, a reliable IC_50_ value could not be determined under the present experimental conditions.

### 2.2. Effect of Isorhamnetin on Steroid Hormone Secretion

**Figure 2:**
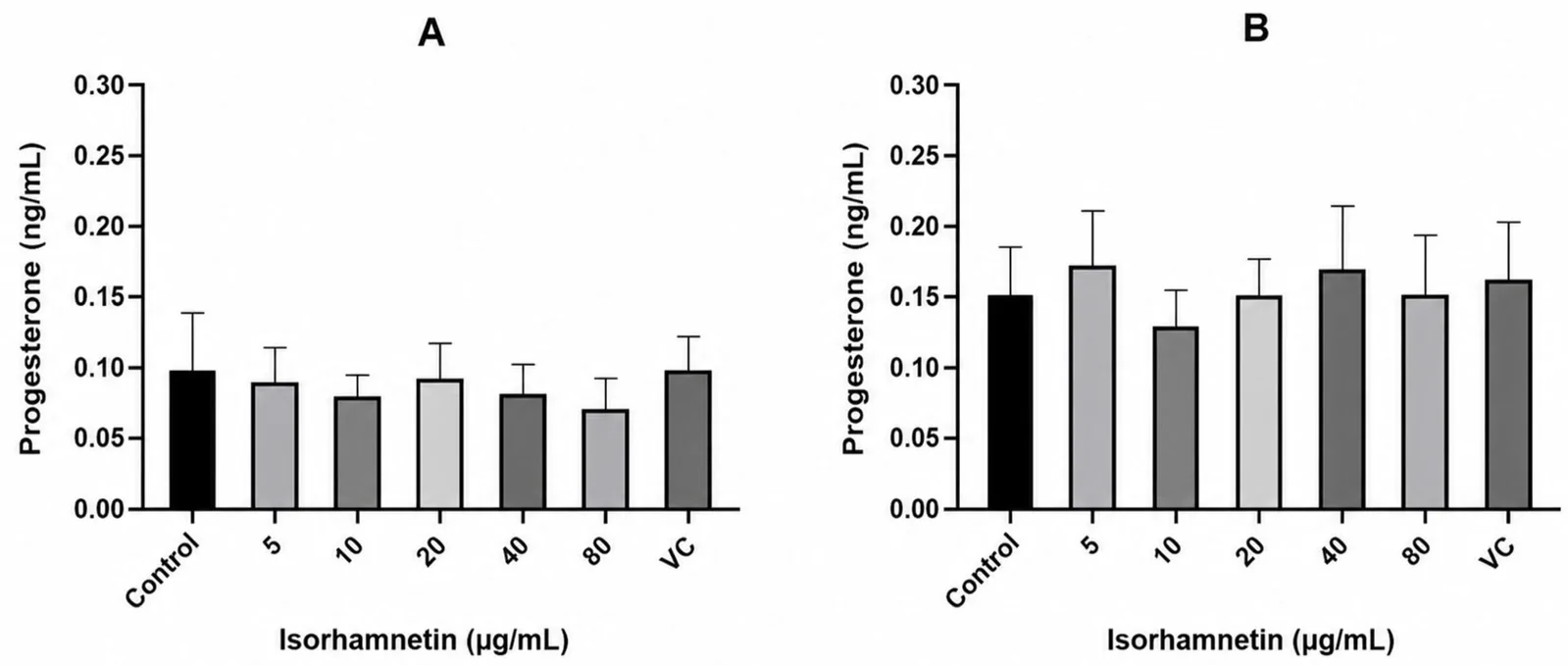
Progesterone secretion by ovarian cells HGL5 (**A**) and COV434 (**B**) after the addition of isorhamnetin in vitro (ng/mL). Cells cultured in medium without additives represent the control group (Control). Cells cultured with ethanol and DMSO in amounts corresponding to the highest applied concentration of the additive represent the vehicle control (VC). Other experimental groups represent cells with selected concentrations of isorhamnetin (5, 10, 20, 40, and 80 µg/mL) for 24 h. Data are expressed as mean values ± SEM. Insignificant differences between the control and experimental groups (*p* ≥ 0.05). Determined by ELISA method.

**Figure 3:**
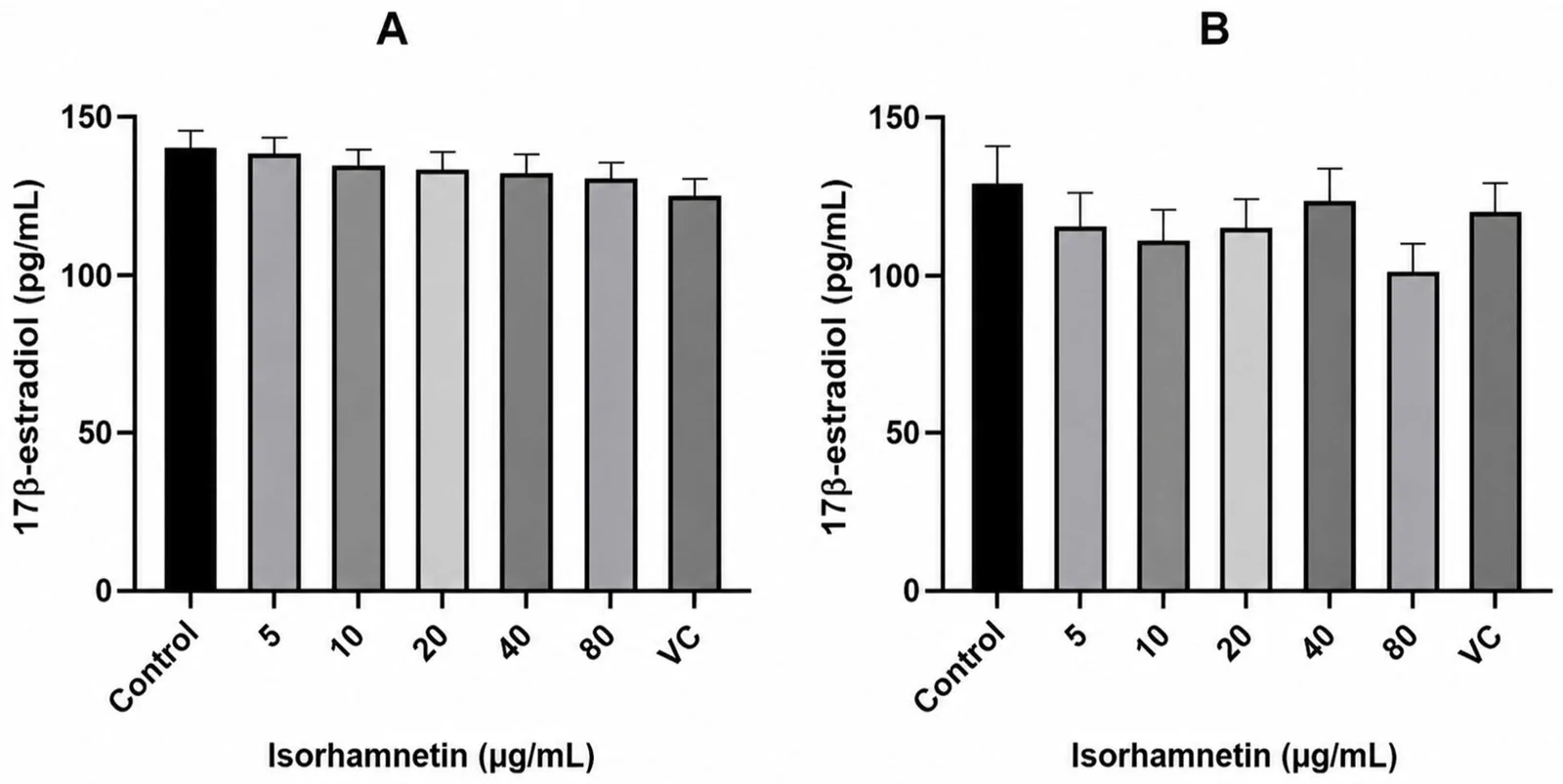
Secretion of 17β-estradiol by ovarian cells HGL5 (**A**) and COV434 (**B**) after the addition of isorhamnetin in vitro (pg/mL). Cells cultured in medium without additives represent the control group (Control). Cells cultured with ethanol and DMSO in amounts corresponding to the highest applied concentration of the additive represent the vehicle control (VC). Other experimental groups represent cells with selected concentrations of isorhamnetin (5, 10, 20, 40, and 80 µg/mL) for 24 h. Data are expressed as mean values ± SEM. Insignificant differences between the control and experimental groups (*p* ≥ 0.05). Determined by ELISA method.

To investigate the effects of isorhamnetin on steroid hormone secretion, progesterone and 17β-estradiol levels were measured in HGL5 and COV434 granulosa cells using ELISA after 24 h of treatment. No significant changes (*p* ≥ 0.05) in progesterone secretion were observed in either HGL5 or COV434 cells compared to control (Figure 2). Similarly, the secretion of 17β-estradiol remained unchanged (*p* ≥ 0.05) across all tested concentrations of isorhamnetin. These results indicate that isorhamnetin did not significantly affect basal steroidogenesis in granulosa cells (Figure 3).

### 2.3. Effect of Isorhamnetin on Secretion of Growth Factors

**Figure 4:**
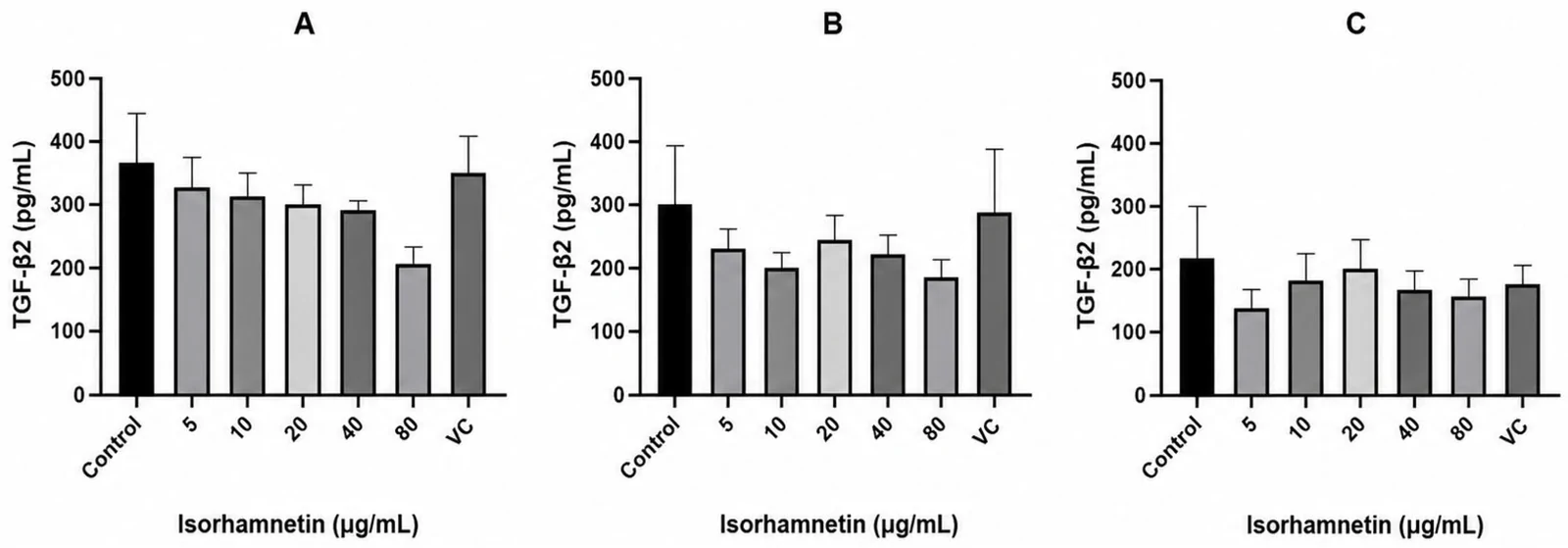
Production of transforming growth factor β2 (TGF-β2) by ovarian cells HGL5 (**A**), COV434 (**B**), and OVCAR-3 (**C**) after addition of isorhamnetin in vitro (ng/mL). Cells cultured in medium without additives represent the control group (Control). Cells cultured with DMSO at a concentration corresponding to the highest applied concentration of the additive represent the vehicle control (VC). Other experimental groups represent cells with selected concentrations of isorhamnetin (5, 10, 20, 40, and 80 µg/mL) for 24 h. Data are expressed as mean values ± SEM. Insignificant differences between the control and experimental groups (*p* ≥ 0.05). ELISA.

**Figure 5:**
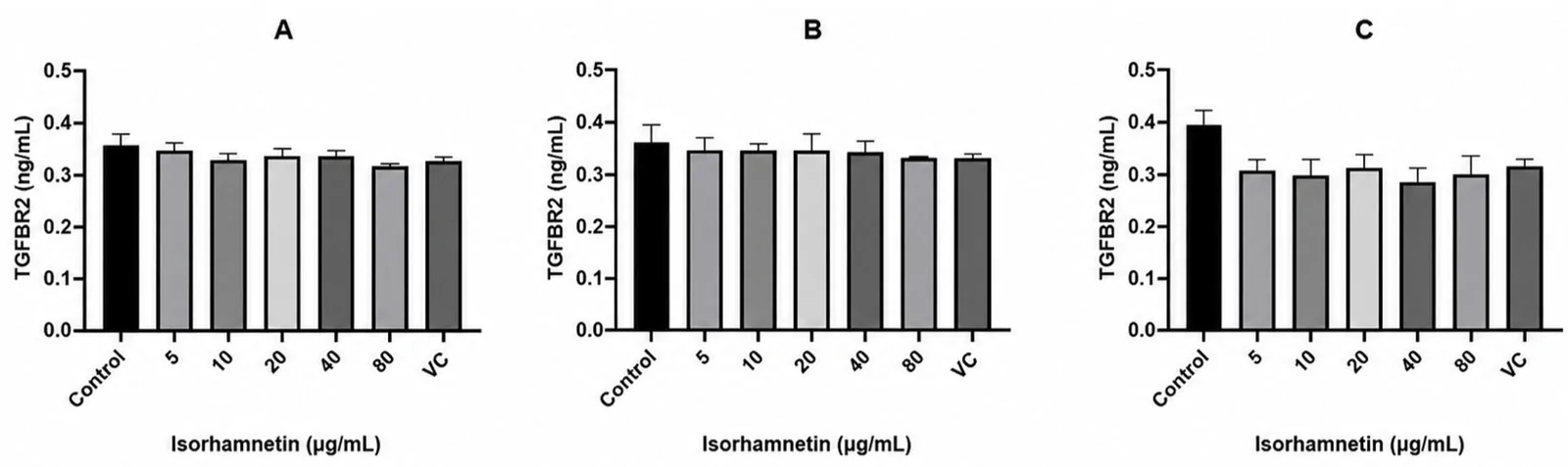
Presence of TGF-β type 2 receptor (TGFBR2) in ovarian cells HGL5 (**A**), COV434 (**B**), and OVCAR-3 (**C**) after adding isorhamnetin in vitro (ng/mL). Cells cultured in medium without additives represent the control group (Control). Cells cultured with DMSO at a concentration corresponding to the highest applied concentration of the additive represent the vehicle control (VC). Other experimental groups represent cells with selected concentrations of isorhamnetin (5, 10, 20, 40, and 80 µg/mL) for 24 h. Data are expressed as mean values ± SEM. Insignificant differences between the control and experimental groups (*p* ≥ 0.05). Determined by ELISA method.

To evaluate the potential modulatory effects of isorhamnetin on growth factor signalling, the secretion of transforming growth factor β2 (TGF-β2) (Figure 4) and the presence of its TGF-β receptor type 2 (TGFBR2) (Figure 5) were assessed in HGL5, COV434, and OVCAR-3 ovarian cell lines. A slight, dose-dependent decrease in TGF-β2 levels was observed in all three cell lines after isorhamnetin treatment, but without statistical significance (*p* ≥ 0.05) compared to controls. Similarly, no significant changes (*p* ≥ 0.05) in TGFBR2 levels were detected in any of the tested cell lines.

### 2.4. Effect of Isorhamnetin on the Presence of AIF

**Figure 6:**
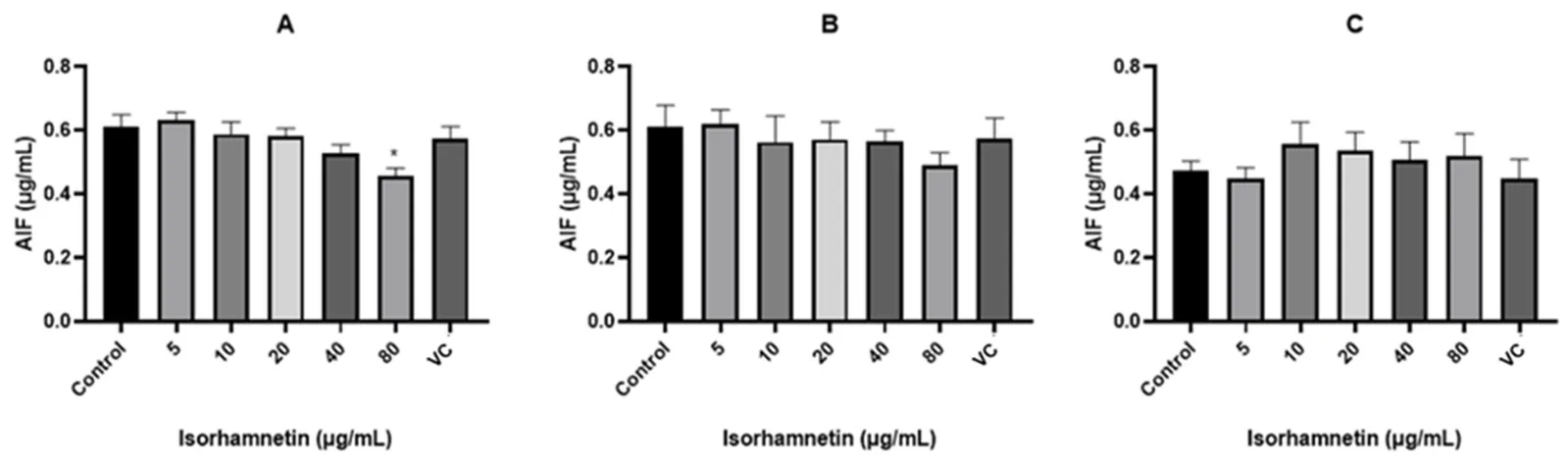
Presence of apoptosis-inducing factor AIF after addition of isorhamnetin in ovarian cells HGL5 (**A**), COV434 (**B**), and OVCAR-3 (**C**) in vitro (µg/mL). Cells cultured in medium without additives represent the control group (Control). Cells cultured with DMSO at a concentration corresponding to the highest applied concentration of the additive represent the vehicle control (VC). Other experimental groups represent cells with selected concentrations of isorhamnetin (5, 10, 20, 40, and 80 µg/mL) for 24 h. The data are expressed as mean values ± SEM. Significant differences between the control and experimental groups are at the * (*p* ≤ 0.05) level. ELISA.

To assess the potential pro-apoptotic effects of isorhamnetin, the presence of apoptosis-inducing factor (AIF) was evaluated in HGL5, COV434 and OVCAR-3 ovarian cell lines. A significant decrease (*p* ≤ 0.05) in AIF levels was observed only in healthy HGL5 cells at 80 µg/mL compared to the control. In contrast, no significant changes (*p* ≥ 0.05) in AIF levels were detected in COV434 or OVCAR-3 tumour cells following isorhamnetin treatment (Figure 6).

## 3. Discussion

The present work provides descriptive evidence of differential responses of ovarian cell lines to isorhamnetin rather than mechanistic insight into its mode of action. Isorhamnetin, a methylated derivative of quercetin, has recently gained attention for its cytotoxic, anti-proliferative, and pro-apoptotic potential in various cancer models [12]. In the present study, we evaluated its effects on ovarian cell lines of different origins—non-tumour granulosa cells (HGL5), granulosa tumour cells (COV434), and epithelial ovarian carcinoma cells (OVCAR-3). The findings revealed a clear dose-dependent reduction in cell viability following isorhamnetin exposure, with the most pronounced effect observed in the malignant cell lines. Viability of OVCAR-3 cells significantly decreased at concentrations as low as 10 µg/mL, whereas a comparable reduction in HGL5 cells was evident only at higher concentrations. This differential response highlights the preferential cytotoxicity of isorhamnetin toward cancer cells, consistent with previous studies demonstrating mitochondria-dependent apoptosis induced by isorhamnetin in malignant models [8,12,14,15].

Mechanistically, isorhamnetin has been shown to activate the caspase-3 cascade, increase the Bax/Bcl-2 ratio, promote cytochrome c release, and cause PARP cleavage, ultimately leading to mitochondrial dysfunction and accumulation of reactive oxygen species [8]. Our findings are consistent with mechanisms reported previously, although these mechanisms were not directly investigated in the present study. Comparable antitumour effects of isorhamnetin were documented in gastric carcinoma [8], endometrial carcinoma [16], and pancreatic carcinoma models [17], reinforcing its anticancer potential. Although ovarian tumour-derived cell lines were more sensitive than the non-tumour HGL5 granulosa cell line, the present study cannot conclude tumour-specific cytotoxicity because only one non-tumour ovarian cell model was included. Therefore, the observed effect should be interpreted as preferential rather than tumour-specific cytotoxicity. Nevertheless, the decreased viability observed in non-tumour granulosa cells at higher doses indicates that concentration and selectivity remain critical parameters when considering the therapeutic application of isorhamnetin.

Steroid hormones, particularly progesterone and 17β-estradiol, are essential regulators of ovarian physiology, produced via the cooperative action of theca and granulosa cells under luteinizing hormone and follicle-stimulating hormone [2,12,14]. In our study, isorhamnetin did not significantly affect the secretion of these hormones in HGL5 or COV434 cells. This observation aligns only partially with the findings of Li et al. (2021) [18], who reported that isorhamnetin modulated ovarian steroidogenesis by promoting estrogen and inhibiting progesterone secretion through regulation of steroidogenic enzymes (CYP19A1, StAR, 3β-HSD) and activation of the PI3K/Akt pathway. That study also described increased expression of proliferative markers (C-myc, cyclins A–E) and reduced apoptosis, suggesting a complex role of isorhamnetin in reproductive cell regulation. Comparatively, the structurally related flavonoid quercetin was shown to reduce proliferation markers and enhance apoptosis in granulosa cells while stimulating 17β-estradiol release and suppressing progesterone production [3]. These findings imply that both flavonoids may influence ovarian hormonal homeostasis through modulation of proliferative and apoptotic signalling cascades, although the magnitude and direction of effects depend on concentration, cell type, and experimental context. However, hormone secretion measured at concentrations associated with substantial loss of viability should be interpreted with caution, since reduced cell viability itself may influence secretory activity independently of endocrine regulation.

Apoptosis-inducing factor (AIF) is a mitochondria-associated protein that triggers caspase-independent apoptosis upon translocation to the nucleus following cellular damage [19]. In the present study, isorhamnetin induced a significant reduction in AIF levels only in non-tumour HGL5 cells at 80 µg/mL, whereas no significant changes were detected in COV434 and OVCAR-3 lines. Literature addressing AIF modulation by isorhamnetin is limited; however, studies on quercetin suggest that similar flavonoids can disrupt mitochondrial integrity, promote AIF release, and activate caspase-3-dependent apoptosis [20]. The observed response in HGL5 cells may thus reflect partial activation of mitochondrial pathways at higher concentrations without full apoptotic commitment in tumour cells.

Transforming growth factor β2 (TGF-β2) and its receptor TGFBR2 play key roles in regulating cell proliferation, differentiation, and immune balance, and alterations in their signalling pathways have been implicated in cancer progression [21]. In our experiments, isorhamnetin induced a non-significant decrease in TGF-β2 and TGFBR2 levels across all ovarian cell lines, suggesting a limited modulatory effect under the tested conditions. This finding contrasts with studies on other plant-derived polyphenols, such as ellagic acid and punicalagin, which were reported to suppress proliferation via the TGF-β/Smad pathway in breast and ovarian carcinoma cells [14,15]. The lack of significant changes in our study may indicate that isorhamnetin does not directly interfere with TGF-β-mediated signalling at the applied concentrations, or that its effects are more pronounced under prolonged exposure.

Our data provide evidence that isorhamnetin exerts a preferential dose-dependent cytotoxic effect on ovarian tumour cells while exerting minimal influence on basal steroidogenesis and growth factor signalling. The observed changes in AIF may indicate partial involvement of mitochondrial pathways. Together with the preferential reduction in tumour cell viability, these findings support further investigation of isorhamnetin as a potential candidate for ovarian carcinoma prevention or adjunctive therapy. However, the concentration-dependent effects on non-tumour cells highlight the need for detailed mechanistic and dose–response studies to ensure safety and efficacy before clinical translation.

## 4. Materials and Methods

### 4.1. Model Cells

#### 4.1.1. Human Granulosum Cell Line HGL5

The immortalized human ovarian cell line granulosum HGL5 (ABM^®^, Richmond, BC, Canada) was cultured in DMEM (Dulbecco’s Modified Eagle Medium, Gibco, Waltham, MA, USA) culture medium with recommended supplements according to the manufacturer’s instructions, supplemented with 10% FBS (Fetal bovine serum; BioWhittaker^TM^, Walkersville, MD, USA) and 1% antibiotic–antimycotic (Sigma, St. Louis, MO, USA). The cells were grown in 75 cm^2^ culture flasks (Nunc^TM^, Roskilde, Denmark) under standard conditions for in vitro culture (37 °C, 95% relative humidity, 5% CO_2_) until 70–80% confluency.

#### 4.1.2. Human Ovarian Granulosa Tumour Cell Line COV434

Immortalized human ovarian granulosa tumour cell line COV434 (ECACC, Salisbury, UK) was cultured in DMEM (Dulbecco’s Modified Eagle Medium, Gibco, Waltham, MA, USA) culture medium with recommended supplements according to the manufacturer’s instructions, supplemented with 10% FBS (Fetal bovine serum; BioWhittaker^TM^, Walkersville, MD, USA) and 1% antibiotic–antimycotic (Sigma, St. Louis, MO, USA). The cells were grown in 75 cm^2^ culture flasks (Nunc^TM^, Roskilde, Denmark) under standard conditions for in vitro culture (37 °C, 95% relative humidity, 5% CO_2_) until 70–80% cell confluence was achieved.

#### 4.1.3. Human Ovarian Carcinoma Cell Line OVCAR-3

The human epithelial cell line of ovarian carcinoma NIH:OVCAR-3 was obtained from the American Type Culture Collection (ATCC^®^ HTB-163^TM^, Manassas, VA, USA) and cultured in sterile RPMI 1640 (Roswell Park Memorial Institute Medium; Gibco-BRL, Rockville, MD, USA) enriched with 10% FBS (Fetal bovine serum; Sigma-Aldrich, St. Louis, MO, USA), 0.5% antibiotic–antimycotic (Invitrogen, CA, USA) and 1% non-essential amino acids (Sigma Aldrich, Gillingham, UK). The cells were grown in 75 cm^2^ culture flasks (Nunc^TM^, Roskilde, Denmark) under standard in vitro culture conditions (37 °C, 95% relative humidity, 5% CO_2_) until 70–80% cell confluence was achieved.

### 4.2. Cell Preparation, Passage and Culture

The in vitro study involved the passage of ovarian cell cultures. The ovarian cell lines HGL5, COV434, and OVCAR-3, which were purchased and thawed, were mixed again with culture medium and placed in culture flasks. They were then cultured under standard in vitro conditions until they formed a continuous monolayer of cells. The pre-cultivation step was followed by trypsinisation and centrifugation (1200 rpm, 5 min). Depending on the specific experiment and type of analysis, the cells were further seeded at a precisely defined concentration into 6/96-well culture plates (Grainer, Frickenhausen, Germany). Before the start of each experiment, an DMSO solution of isorhamnetin (Sigma-Aldrich, St. Louis, MO, USA) was prepared and diluted to the appropriate concentrations in the culture medium. Depending on the experimental groups, the cells were incubated without addition (Control) or with the addition of isorhamnetin at concentrations of 5, 10, 20, 40, and 80 µg/mL for 24 h. Vehicle control (VC) consisted of DMSO at the same final concentration as that used for the highest isorhamnetin concentration. After incubation with the additives, the cells were tested for viability (AlamarBlue^TM^ test). The medium and cell lysates were used for selected immunological analyses (ELISA; steroid hormones, growth factors and their receptors, and selected markers of apoptosis).

### 4.3. Laboratory Methods Used

#### 4.3.1. AlamarBlue Test

Cell viability was assessed using the AlamarBlue^TM^ test (BioSource International, Nivelles, Belgium) as a suitable indicator of cell health, metabolism, and viability. This colorimetric method uses the indigo blue reagent resazurin as an oxidation-reduction agent, which, depending on cell metabolism, is converted into the highly fluorescent pink product resorufin. A volume of 100 µL of cell suspension with a cell concentration of 1 × 10^4^·mL^−1^ was added to 96-well culture plates and cultured with experimental concentrations of extract. AlamarBlue^TM^ reagent was added to all wells of the plate in a volume of 10 µL 4 h before the end of incubation. For each experiment, empty wells without cells containing only culture medium with AlamarBlue^TM^ solution were prepared as a negative control. AlamarBlue^TM^ reduction was measured spectrophotometrically (ELISA reader, Multiskan FC, Thermo Fisher Scientific, Vantaa, Finland) by recording the absorbance at an excitation wavelength of 560 nm and an emission wavelength of 590 nm. The result of the effect of multiple metabolic reactions was then expressed as a percentage reduction according to the manufacturer’s instructions [14,15].

#### 4.3.2. ELISA

The effects of isorhamnetin on cell secretory activity were evaluated using the ELISA immunological method. Cells were seeded onto 6-well culture plates at a concentration of 2 × 10^5^ cells/mL and cultured with additives. Using an ELISA kit and following the manufacturer’s instructions, the release of steroid hormones (progesterone, 17β-estradiol) was determined by analyzing the culture medium. Steroid receptors, growth factors and their receptors, as well as markers associated with cell apoptosis, were analyzed from cell lysates. The principle of the ELISA method is based on the competition of the antigen (hormone, growth factor, etc.) contained in the sample with the conjugate (horseradish peroxidase—HRP) for a limited site by binding to the appropriate specific antibody (IgG), which is pre-coated on 96-well microtiter plates (MTP, Grainer, Frickenhausen, Germany). After the required incubation (37 °C), the excess conjugate was removed by washing, and then the base substrate (H_2_O_2_, TMB 0.25 g·L^−1^) was added to each well of the plate. After the recommended time for maximum colour development, the enzymatic reaction was terminated with a stopping solution (sulphuric acid 0.15 mol·L^−1^). Finally, spectrophotometric measurement of sample absorbance was performed according to references at a wavelength of 450 nm on a microplate ELISA reader (Multiskan FC, Thermo Fisher Scientific, Vantaa, Finland). The concentration of the monitored indicator in the sample was expressed based on the optical density of the calibration solutions.

## 5. Conclusions

The present study suggests the biological activity of the flavonoid isorhamnetin and its potential modulatory effects on ovarian cell function in vitro. Isorhamnetin exerted a dose-dependent cytotoxic effect, significantly reducing the viability of ovarian tumour cells (COV434 and OVCAR-3) at relatively low concentrations while affecting non-tumour granulosa cells (HGL5) only at higher doses. This preferential response indicates its potential role as an inhibitor of ovarian tumour cell proliferation. Moreover, isorhamnetin did not significantly alter the secretion of steroid hormones (progesterone and 17β-estradiol) or the expression of TGF-β2 and TGFBR2, suggesting that its biological activity may involve alternative apoptotic mechanisms. A reduction in apoptosis-inducing factor (AIF) observed in HGL5 cells at the highest concentration may indicate partial involvement of mitochondrial pathways.

Taken together, these findings point to isorhamnetin as a promising bioactive compound with potential applications in the prevention or adjunctive management of ovarian cancer. Its suggested antiproliferative and pro-apoptotic properties, together with limited effects on non-tumour granulosa cells within most of the tested concentration range, underline its relevance for future nutraceutical and phytotherapeutic development. These findings provide preliminary evidence supporting further preclinical investigation of isorhamnetin. Nonetheless, additional studies are required to elucidate its molecular mechanisms, optimize dosing, and evaluate its synergistic potential with other phytonutrients using biotechnological approaches aimed at improving bioavailability and efficacy in food science, pharmacology, and biomedicine.

### Study Limitations

The present study has several limitations. First, only selected functional endpoints were evaluated, whereas key molecular pathways associated with apoptosis, oxidative stress, and proliferation were not investigated. Therefore, mechanistic conclusions cannot be drawn. Second, only immortalized ovarian cell lines were used, limiting extrapolation to in vivo conditions. Third, only a single exposure period (24 h) was evaluated. Finally, no biological positive controls were included for steroidogenesis or TGF-β signalling, which should be considered in future studies.

## Data Availability

The data presented in this study are available from the corresponding author upon reasonable request.

## Abbreviations

The following abbreviations are used in this manuscript:

AIF	Apoptosis-Inducing Factor
AKT	Protein Kinase B
ATCC	American Type Culture Collection
CO_2_	Carbon Dioxide
COV434	Human Granulosa Tumour Cell Line
DMEM	Dulbecco’s Modified Eagle Medium
DMSO	Dimethyl Sulfoxide
ECACC	European Collection of Authenticated Cell Cultures
ELISA	Enzyme-Linked Immunosorbent Assay
FBS	Fetal Bovine Serum
HGL5	Human Granulosa Cell Line
HRP	Horseradish Peroxidase
IgG	Immunoglobulin G
MAPK	Mitogen-Activated Protein Kinase
MTP	Microtiter Plate
NF-κB	Nuclear Factor Kappa B
NIH	National Institutes of Health
OVCAR-3	Human Ovarian Carcinoma Cell Line
PARP	Poly(ADP-ribose) Polymerase
PI3K	Phosphoinositide 3-Kinase
RPMI 1640	Roswell Park Memorial Institute 1640 Medium
SEM	Standard Error of the Mean
StAR	Steroidogenic Acute Regulatory Protein
TGF-β2	Transforming Growth Factor Beta 2
TGFBR2	Transforming Growth Factor Beta Receptor Type 2
TMB	3,3′,5,5′-Tetramethylbenzidine
